# “I Don’t Have Any Limits”: A Qualitative Analysis of Individual Gambling Self-Control Strategies

**DOI:** 10.3390/ijerph21111401

**Published:** 2024-10-23

**Authors:** Emily Nolan, Rebecca Scheurich, Tara Hahmann, Adèle Morvannou, Emilie Y. Jobin, Eva Monson

**Affiliations:** 1 Faculty of Medicine and Health Sciences, Université de Sherbrooke, Sherbrooke, QC J1H 5N4, Canada; emily.nolan@usherbrooke.ca (E.N.); rebecca.scheurich@usherbrooke.ca (R.S.); adele.morvannou@usherbrooke.ca (A.M.); emilie.jobin@usherbrooke.ca (E.Y.J.); 2 MAP Centre for Urban Health Solutions, St. Michael’s Hospital, Toronto, ON M5B 1W8, Canada; tara.hahmann@unityhealth.to

**Keywords:** gambling, self-control strategies, qualitative, problem gambling

## Abstract

Despite existing knowledge on self-control strategies in the context of problem gambling, further insight is needed to understand a broader spectrum of self-control strategies among individuals who span the continuum of problem gambling. This qualitative study drew on the experiences and perceptions of individuals engaging in recreational gambling as well as those at the at-risk and problem gambling levels to explore various self-control strategies and their nuances. Thirty semi-structured interviews, guided by open-ended questions exploring how gamblers define and practice responsible gambling and their understanding of responsible gambling interventions, were conducted in Quebec, Canada. Thematic analysis identified three main themes: setting limits on frequency, time, and spending, playing smart (i.e., mindful gambling), and recognizing strategy limitations. Despite employing various strategies, participants struggled to maintain self-control. Maintaining self-control was particularly difficult for those with higher Problem Gambling Severity Index scores. These findings underscore the complexities of managing gambling behavior and, more specifically, these findings contribute to understanding the role of self-control in mitigating gambling problems. This study highlights the need to focus on developing comprehensive support systems and harm minimization measures within gambling environments to better support individuals across the gambling spectrum.

## 1. Introduction

Gambling-related harm has emerged as a recognized public health concern [1,2,3,4]. Gambling-related harm is a broad term used to describe negative consequences related to gambling activities and can include financial losses, accumulation of debt, increased crime, emotional hardship, and exacerbation of existing mental health issues [5]. These consequences affect not only those who gamble but can also extend to family, friends, and the individual’s community. According to several studies, the harm associated with gambling is comparable in size to the harm associated with major depressive illness or alcohol use disorder and dependency [6].

Given the impact of these harms, the government and gambling industry promote what responsible and rational engagement with gambling should look like, focusing on personal responsibility (e.g., adjusting gambling habits, exerting control, employing self-monitoring behaviors) [7,8]. With messaging focused on individual control, a small body of research has investigated the self-control strategies employed by those who gamble [9,10,11]. Subramaniam and colleagues (2017) reported various cognitive and behavioral strategies used to prevent problem gambling among a sample of older adults in Singapore, including avoiding cognitive distortions, setting limits, and not gambling alone [10]. Several studies have found that both people who do not experience problem gambling and those who may be experiencing problem gambling used similar responsible gambling strategies, including setting time and money limits, arriving with a set amount of money, and leaving sources of money at home [10,11]. A more recent study by Flores-Pajet and colleagues explored additional self-control strategies in addition to monetary-focused strategies, such as limits on frequency and time, among a sample of those at low to moderate risk of experiencing problem gambling [9]. They noted that these additional strategies were only discussed when probed by interviewers rather than being readily discussed, as was the case with monetary limits.

Although people who gamble will try to use self-control strategies, some have trouble adhering to them. One study by Currie and colleagues of Canadian adults who reported gambling in the past 12 months showed that around 45% of participants were not able to adhere to the limits (e.g., monetary, frequency, time) they imposed on their gambling activities [12]. Other studies have also demonstrated that individuals, whether they experience gambling problems or not, have trouble sticking to their self-imposed gambling limits [13,14]. It has been noted that adherence is particularly difficult when more temptations (e.g., food, alcohol) are present [9,14]. Yi and colleagues’ paper suggested that managing emotions and coping with stress may take precedence over adhering to gambling limits, potentially leading individuals to prioritize immediate gratification, such as indulging in tempting foods, over other goals, like adhering to gambling limits [14]. As well, Flores-Pajet and colleagues’ study highlighted that substance use (e.g., alcohol, cannabis) influenced adherence to these self-control strategies [9].

The importance of self-control strategies in managing gambling behavior has been emphasized by the gambling industry, and some research has identified mainly monetary along with some other self-control strategies, including time and frequency limits, as the primary strategies individuals will use. Despite the existing knowledge on self-control strategies and their efficacy, more insight is needed to better understand a wider spectrum of self-control strategies among individuals who span the continuum of problem gambling. This qualitative study drew on the experiences and perceptions of those who gamble recreationally and at the at-risk and problem levels to explore a broader spectrum of self-control strategies while tapping into additional nuances, such as the complex dynamics between strategies, the need to engage in mindful play by way of strategic decision making, and perceived adherence to and efficacy of strategies, including their limitations.

## 2. Materials and Methods

This qualitative exploratory study draws on analyses of 30 semi-structured interviews that were part of a descriptive qualitative study on the conceptualization of responsible gambling among a population of adult gamblers in Quebec, Canada. This mode of inquiry centers directly on the lived experience of those for whom interventions are designed and developed, which allows for a more direct understanding of the perceived effectiveness of responsible gambling discourse, initiatives, and tools [15]. Ethical approval was obtained from the Comité d’éthique de la recherche des Lettres et Sciences Humaines of the Université de Sherbrooke (N/Réf. 2018-1751/Monson) before the study began.

### 2.1. Recruitment and Participants

Participants were recruited using advertisements in Montreal (free-of-cost newspapers, libraries, community organizations, social media, bars, and convenience stores). Potential participants were also encouraged to share information about the study with peers who may also fit the inclusion criteria. To meet the inclusion criteria for the study, participants had to have gambled in the past 12 months, be 18 years of age or older, and be able to complete an interview in English or French.

Prospective participants completed a screening questionnaire to assess their eligibility, which included sociodemographic and gambling practices questions. The Problem Gambling Severity Index (PGSI), a subsection of the Canadian Problem Gambling Index [16], was administered as part of the screening questionnaire. This 9-item, standardized, self-report screening instrument was designed to evaluate problem gambling behaviors and consequences experienced by individuals over the past 12 months. Each item is scored using Likert response categories (0 = never, 1 = sometimes, 2 = most of the time, 3 = almost always). Items are summed and used to establish 4 groups that reflect an increasing severity of problem gambling (non-problem gambling = 0, low risk = 1 to 2, moderate risk = 3 to 7, and problem gambling = 8+). Research has shown that the PGSI demonstrates adequate internal validity and reliability [17]. Informed consent was obtained from each participant before the interview began. Participants were informed of the study’s purpose, procedures, risks, and benefits, confidentiality, compensation, and the voluntary nature of their participation.

### 2.2. Data Collection

Data collection took place between February and March 2020. Each participant completed an individual, in-person, semi-structured interview (ranging in duration from 22 to 85 min, median length of 50 min) administered by a trained research coordinator. The semi-structured interview guide, which included 5 open-ended questions, was developed by the research team. The questions explored how participants define and practice responsible gambling as well as their understanding of responsible gambling interventions, such as “Do you know of any existing responsible gambling strategies?” Specifically, the interviews explored definitions/interpretations of responsible gambling, participants’ current responsible gambling strategies, barriers to using responsible gambling measures, motivations for gambling, and the ways in which responsible gambling is enacted and practiced by participants. As remuneration for their time, participants were given CAD 25 gift certificates.

A total of 30 interviews took place. The sample included 20 men and 10 women ranging in age from 25 to 75 years old (M = 50.5 years). When asked which gambling activities participants engaged in, lottery tickets (n = 16) and electronic gambling machines (EGMs; n = 15) were the most popular forms of gambling, and most participants reported multiple gambling activities. A detailed description of the sociodemographic characteristics of the participants can be found in Table 1.

### 2.3. Data Analysis

Interviews were audio-recorded and later transcribed verbatim and anonymized. Each digital audio recording was listened to multiple times, including for the initial transcription by the transcriber and again by the original interviewer to ensure accuracy. Transcripts were then imported into NVivo 10 software. Using thematic analysis as a framework [18], the transcripts were read and transposed into themes representative of the analyzed content. In parallel with the analysis, reflective memos were written to document ideas about the patterns and relationships observed across themes. The themes identified were tagged alongside the text and were then grouped, combined, and ordered into central themes [19].

## 3. Results

Discussions of self-control in the context of gambling resulted in three main thematic categories: (1) setting various limits, monetary and otherwise; (2) playing smart, encompassing strategies around remaining aware and mindful of gambling play; and (3) limitations of the strategies identified by participants and the importance of external support in helping to adhere to them.

### 3.1. Limiting Strategies

#### 3.1.1. Limits on Frequency, Time, and Spending and Their Interactions

In discussing self-control strategies, participants emphasized the importance of implementing limits to mitigate negative consequences associated with gambling, whether they be on frequency of play, time spent playing, or spending. These limits took various forms, from setting a cap on the number of gambling sessions per week to allocating a specific amount of time for each session. Additionally, individuals often established spending limits, such as budgeting their gambling activity and allocating a predetermined sum for each outing. However, the delineation between these limit types was not always clear-cut, and there existed room for them to overlap, leading to a potential interaction between frequency, time, and spending limiting strategies.

*Frequency and Time.* Participants discussed limiting how often they engage in gambling activities, meaning they would set intervals for the frequency at which they should play. Limits were often decided by the individuals themselves, with no mention of specific reasoning behind their choices. The most common strategy amongst participants was to limit gambling sessions to weekly or monthly outings:

*“Also try to limit how often you go and try to say to yourself, for example, “I’m not going more than once a month.””*

*(Dominic, age 28, PGSI 9)*

Compared to the most common frequency and time strategies of imposing weekly or monthly limits, several participants mentioned less specific methods of limiting the frequency of gambling activities by trying not to engage in the activity “all the time”. One participant described how long-term abstinence was not realistic, but, for shorter periods of time, it could be a good strategy to implement:

*“Okay, one, short-term abstinence, yes, that can be good. Because, you know, the new fashion is intermittent fasting. Well, that’s it. You could say “okay we, we’re going to do a fast of, let’s say, you know, I don’t buy any or every other week”. Right, yes. That could be good.”*

*(Elizabeth, age 46, PGSI 2)*

Respecting time limits was a lesser-employed method. Compared to other self-control strategies, imposing a time limit was only mentioned by one participant:

*“But every time I go somewhere, I put limits, I put limits, I said “I’m going to stay an hour,” “I’m going to stay thirty minutes,””*

*(Sabrina, age 47, PGSI 10)*

*Spending*. Many participants discussed financial limiting strategies, with a prevalent strategy involving budgets. Some spoke about how they tailored their budget to individual sessions or specific time frames. These budgets were diverse, often reflecting the unique financial circumstances of each participant. Some individuals prioritized creating a financial cushion within their budgets to mitigate potential losses.

Budgeting often involved setting limits for a specific period (e.g., a session, week, or month) by determining the allocated amount of money for gambling during that time. A few participants mentioned the concept of budgeting generally without expanding on how they determined their budget and for what time interval it was for. The most common strategies mentioned were setting aside a pre-determined budget per session or per week:

*“But when I go like to the races, I go with a friend and we share 30 bucks each, which means we put our 30 bucks each, that gives us 60 dollars.”*

*(Louis, age 72, PGSI 0)*

*“You set yourself a precise budget and then as soon as you exceed your budget, well, you stop gambling. I’ll give you an example: when I went on vacation in the fall, I went to the casino, I set myself a budget of fifty U.S. dollars for the week, and the moment I exceeded that budget, well, it’s like “we’re quitting”!”*

*(Noelle, age 56, PGSI 0)*

In the context of choosing their budgets, one participant voiced how the choice was often very individualized and how the amounts varied by person:

*“But, like every individual, we don’t have the same budget, we don’t have the same reality, we don’t have the same spending capacity, it’s all relative.”*

*(Olivia, age 48, PGSI 10)*

Others expressed how the amount chosen was in line with what they were willing to lose or deemed appropriate to spend. For some, ensuring there were no financial consequences to their gambling expenses was how their budget was decided:

*“No, and it also depends on the price I’m told. “Listen, we’ll take, we’ll take tickets that cost a hundred bucks a ticket”, I don’t get on board. It’s not in my budget. […] it’s, it’s determining an amount I’m prepared to lose and then once it’s lost, it’s over.”*

*(Joseph, age 66, PGSI 1)*

*“Oh, my budget! Let’s say, I’ll say a hundred dollars, so if it’s something I know I can lose, then there won’t be any financial consequences for me. So when it comes down to it, being responsible, you know, two questions before, it comes down to not, how shall I put it, not putting yourself in a financial bind.”*

*(David, age 43, PGSI 3)*

More specifically, when determining budgets, some participants shared the importance of setting aside money for basic needs, such as groceries, rent, and other day-to-day expenses, before determining the amount to be used for gambling. Strategies would then entail a measured approach to spending where money was set aside for “rainy days” and what was remaining could be used for hobbies, including gambling:

*“It’s certain that we all have expenses, we have, we have things to... so we have to be very careful we have, not to fall into that, then avoid the, avoid spending money before that then, we have to prioritize expenses then, if we have money left over, well maybe we can go and have fun at the casino maybe…”*

*(Jamie, age 36, PGSI 4)*

*“If you have a budget for gambling, you have a budget for your rent, your electricity then, you have to prioritize it too! Yes, yes, you have to prioritize! […] Well, let’s put a roof over your head, electricity, your food, that’s really a priority. You know, after that, what becomes superficial is this, having dinner at a restaurant, playing games.”*

*(Olivia, age 48, PGSI 10)*

*“I always bring myself a certain amount of money when I go to the casino and there’s no way I’m going to take another penny out. I tell myself I’ve played what I had to play, that’s enough. I’m not going to start (using) next week’s grocery shopping just to win.”*

*(Greg, age 59, PGSI 0)*

Participants described that having direct access to cash was associated with increased temptation to gamble, leaving some to employ additional self-control strategies around money. That is, some participants discussed limiting how much money they could access. This strategy took various forms, from depositing money directly into a bank account, whether their own or a family member’s, to restricting the amount readily available for gambling (e.g., depositing in a savings account rather than their chequing account). Others opted to limit the physical cash they brought to the casino, thereby reducing the temptation to indulge in excessive spending while gambling. Some individuals redirected their financial resources towards other endeavors, such as investments. These approaches demonstrated the efforts individuals made to regulate the use of money or credit. For example, one participant described the need to distance themself from money to avoid the temptation to gamble with it:

*“I went to the bank the other day […] I wanted to deposit the 100$, because I don’t want it next to me. I’m like that. I’ll put a lot of obstacles in front of me for my money.”*

*(John, age 51, PGSI 5)*

There was a consensus among some participants regarding the prudent use of credit cards and bank cards, emphasizing the importance of leaving them at home to deter impulsive spending during gambling sessions. These practices were emphasized as helpful steps to avoid giving in to impulses and to exercise responsible financial management:

*“But now, even I don’t even bring my debit card with me anymore, just in case. I keep twenty bucks in my wallet, that’s it. My debit card at home all the time.”*

*(Ben, age 28, PGSI 19)*

Financial self-control strategies for a few also included reorienting their use of money and strategizing to put the money they would have spent on gambling activities towards other things, such as savings or investments. When discussing this strategy, one participant mentioned a preference for setting money aside for savings, highlighting the positive outcome observed:

*“When you lose twice, let’s say two hundred, that’s enough, I don’t want to lose. I’d rather put some aside and save now. […] I finally went a year without taking any. […] But now it’s like I tell you, Loto-Max sometimes it tempts me, or when I see that its my numbers I like to put, well I’ll take a chance. But after that I say to myself no, I look at the next day, all the money I saved in the year anyway, it makes me feel good.”*

*(Gabriella, age 63, PGSI 7)*

*Interactions of Limits*. For some, the breaching of one limit type often triggered a ripple effect, necessitating adjustments in other limits. For example, exceeding a predetermined budget would require the frequency of gambling to be reduced. This highlighted the subtle connection whereas the non-observance of one limit inevitably impacted another. As one participant explained, exceeding their predetermined budget would result in the need to adjust the next time they visited the casino:

*“Well, basically, that’s it, I, I stick to my budget. Then if I see that I’ve gone over, well the next time I go, it’s going to be in a longer time. You know, I’m going to, I’m going to put it off a little longer.”*

*(David, age 43, PGSI 3)*

Another participant mentioned that their monthly budget revolved around one planned outing to the casino per month, emphasizing that increasing the frequency of visits would disrupt the allocated funds they have set aside:

*“But I bring sixty bucks. Then that’s the most I bring each time, you know, like, once a month. I can’t go like three times a month, or I’d be under budget. […] You know, I’d be kind of hard-pressed to buy food, anything.”*

*(Gabrielle, age 53, PGSI 6)*

#### 3.1.2. Limiting Access to Triggers

The context in which individuals gamble was as important as other self-imposed limits. Strategies around controlling triggers to optimize gambling self-regulation included limiting access to specific game types, people, and alcohol.

*Specific Game Types*. Certain locations, such as bar restaurants, were known to have specific games, which were thought to be a trigger or risk factor for some. To avoid access to game types, one strategy was to physically limit oneself from the location where the game type was located. Some identified EGMs specifically as high-risk for uncontrolled play, prompting them to avoid locations where they knew these machines would be present:

*“Well (a strategy I use is), it’s to stay away from temptation first. You stay away from that. You go to a restaurant, you just go to a restaurant, not a bar restaurant. If you’re really sensitive to machines, as soon as you see a machine and then you, in your head, it comes to you, start you, then you try to stay away from that.”*

*(Olivia, age 48, PGSI 10)*

When asked about how to avoid gambling harms, some participants noted the importance of protecting oneself from highly addictive or especially harmful forms of gambling, such as online gambling:

*“I try to avoid online games. From my point of view, I think there is more risk of developing an addiction with online games. The games with the machines and everything. So, to try to control that.”*

*(George, age 38, PGSI 5)*

While avoiding locations might be effective in the offline context, online forms of gambling create new challenges. Some forms of gambling and related activities are easily accessible online, which prompted some participants to install parental controls on their electronic devices to block access:

*“And then... blocking computer games…parental controls, stuff like that.”*

*(Mason, age 58, PGSI 11)*

*Specific People*. Those who have experienced problem gambling struggled to control their gambling when alone. A strategy for some was to gamble with others to avoid losing control. In discussing the challenges of self-control in gambling, one participant emphasized the role of companionship in managing their behavior, highlighting that gambling with others provided a form of external control:

*“I’ve tried to control it, as I’m telling you, I’ve tried to limit it at the casino. Of course, at the casino, I never go on my own, so I go with a friend, so it’s easier to be controlled, because if it’s not me who says get out, there are three or four others who’ll say “let’s go”, so I’ll be out.”*

*(Sabrina, age 47, PGSI 10)*

Avoiding people who might negatively influence gambling behavior was another strategy some employed. One participant mentioned how it was better to refrain from associating with those who gamble, as those relationships potentially encourage gambling behavior:

*“And then avoid meeting people who play. […] When you know someone gambles, other gamblers are attracted to that. […] Like me, when I was consuming, well, it seemed like everybody I met was just consumers. So in the game it’s the same thing.”*

*(Mason, age 58, PGSI 11)*

Another participant spoke to how they decided to cut ties with all the people they viewed as being a bad influence:

*“Then I don’t need to, you know, I cut off all the bad influences that, that surrounded me, all the people that brought me problems or brought me drugs or did fraud, whatever, anything, there, I cut it all off. I blocked Facebook, I don’t talk to that world anymore. I don’t want anything to do with that world. I’ve even changed my cell phone number so they don’t call me, because I don’t want them...”*

*(Ben, age 28, PGSI 19)*

*Alcohol*. A few participants spoke to concurrent alcohol use and gambling, noting that it was said to increase the negative effects of each behavior. Alcohol, impairing cognitive functions and self-control, amplified impulsive gambling tendencies and increased the risk of financial loss or addictive behaviors. For instance, one participant’s experience of consistently losing control while drinking during gambling sessions highlighted the challenge of maintaining effective self-regulation in such situations, prompting the adoption of alcohol avoidance as a strategic response to mitigate these risks:

*“But when you drink alcohol, you become like less responsible. You become like “ah!”, there, you know, you start to be on the party, you feel like playing, you feel like having fun, “ah, I lost a hundred bucks...ah, it’s not a big deal, it’s a hundred bucks, that, we’re going to keep playing”. But it’s like that all the time, it’s, the alcohol, it’s not good when you play.”*

*(Ben, age 28, PGSI 19)*

### 3.2. Play Smart: Knowledge, Skill, and Chance

Beyond setting limits or controlling the conditions within which they played, participants employed a variety of alternative strategies to navigate the uncertain landscape of chance. Key among these is the concept of remaining aware and “playing smart”, which encompassed the mindful management of winnings, the use of both knowledge and skill to minimize risks, and the acknowledgment of the inherently chance-based nature of gambling outcomes. Individuals spoke about strategies they implemented themselves but also spoke more generally about those that were potentially helpful in regulating one’s gambling behavior.

Participants noted that it is often better to stop gambling when you win during a gambling session rather than continuing to play and risking everything. They often tried to reason with themselves about when to stop. In discussing the risks associated with gambling, participants highlighted the need for individuals to discern when their gambling habits might lead to negative outcomes. For example, one participant pointed out instances where players might dangerously escalate their bets, illustrating the importance of self-awareness in recognizing potential consequences:

*“Well... When it gets dangerous (is when you have to withdraw from gambling), when you’re not sure of yourself. You know, you play with fire too much and then... Anyway, when you make $100,000, I’ll be gone. The guy, he goes up to $500,000, then he gets lucky, he pushes his luck. Then he says: “Oh well, I’ll put back another $500,000. It’ll double to a million.” Then he loses it all because... maybe it’s blackjack 21, then they changed your card then the dealer, he’s a cheater, you don’t know that.”*

*(William, age 55, PGSI 3)*

Although participants engaged in a wide array of gambling activities, with most favoring games of chance, many reflected on the need to be methodical in their approach to the game. For participants, this meant that one must educate themselves on the rules of the game and employ specific strategies that are rooted in logic:

*“Poker, then blackjack, you have to practice with friends for at least a year. Then, you’ll be able to decode all your friends’ tricks and nervous tics.”*

*(William, age 55, PGSI 3)*

Another participant remarked that one had to play intelligently, which meant playing within the parameters of both skill and luck:

*“Intelligently means knowing how to play well, even if you always say “it’s just chance”, there’s still a logic to it. […] It’s knowing when he’s got a turned card, let’s say he’s got a face, then you’ve got a face, then you’ve got let’s say fifteen, well then, you can take the chance to go on. Because he can have it one way or another, with a face, you know. You know, you have to, there are probabilities, that’s for sure, you also have to know, you also have to know how to play intelligently. Smart means knowing the game.”*

*(Gabrielle, age 53, PGSI 6)*

Some participants thought that to ensure measured play they needed to remain mindful of the chance-based nature of most games. One participant spoke of gambling in the context of external control, in that luck dictated a win:

*“Actually, it’s a matter of personal conviction. I used to say to myself all the time, if you’re lucky, you’re lucky. If you’re not lucky, you’re not lucky. Whether you buy a thousand tickets or one ticket, it’s pretty much the same thing.”*

*(George, age 38, PGSI 5)*

Even when speaking of games that were more skill-based, some participants noted that games are mainly about luck. That is, even in games of skill, it was important to remain mindful of one’s lack of control over gambling outcomes. In line with this reasoning, one individual voiced the importance of remaining realistic given that gambling activities have this element of chance:

*“Don’t get too excited, also, stay realistic (when gambling). Well, knowing that our chances of winning are minimal. And then making sure that it [gambling] doesn’t take up a very large part of your life. Not to become addicted, if I can say that, or dependent on that kind of activity to be satisfied or fulfilled, I would say.”*

*(Anthony, age 53, PGSI 9)*

### 3.3. Limitations of Self-Control and Importance of External Supports

While the above-noted strategies were employed to regulate gambling behavior, when asked about their ability to employ these strategies, many revealed they were unable to harness self-control. When probed, participants divulged that these strategies are often ineffective, suggesting the need for external controls or interventions.

Despite a plethora of individual self-control strategies reported—such as restricting access to funds or imposing time limits—most participants found them insufficient for fostering self-control. Gamblers with higher PGSI scores articulated the challenges they faced in implementing these strategies, shedding light on the limitations of self-imposed measures in curbing gambling behaviors:

*“Me, that’s why I know I’ve got a gambling problem, I don’t, I have trouble like, setting a limit for myself, well, I don’t have any limits.”*

*(Ben, age 28, PGSI 19)*

Many individuals, particularly those with high PGSI scores, expressed their struggle with adhering to these strategies, citing a lack of self-control as a significant barrier:

*“So I’ve tried to control myself, to be responsible, but I can tell you in the case of 75% of the time, I can’t.”*

*(Sabrina, age 47, PGSI 10)*

*“Me, I’m the first person, I don’t, I’m not a responsible gambler, because if I would have to be responsible, I wouldn’t bring my bank card. I would take a hundred bucks and then leave my bank card at home, but that never happens. So I take my bank card all the time and then I take another hundred and fifty, two hundred bucks all the time and then I know I’m not a responsible player.”*

*(Ben, age 28, PGSI 19)*

While self-control strategies were implemented, adherence to them became difficult when addiction took hold. A few participants drew parallels between problem gambling and substance addiction. Two participants discussed how gambling addiction was as severe as a dependency to cocaine:

*“It was addictive, it was, it was like cocaine basically, it was the same thing there. It’s, it’s a drug, it’s one of the strongest drugs plus (equivalent to) gambling. It is, it is an addiction, it is, it is serious, there!”*

*(Ben, age 28, PGSI 19)*

*“It’s the same as coke, it’s the same as poker machines. You try coke and the poker machine, it does exactly the same thing.”*

*(Louis, age 72, PGSI 0)*

When struggling with gambling addiction, engaging in gambling practices that prioritize moderation and restraint became possible only when individuals could exert adequate self-control. However, once an uncontrollable urge to gamble set in, maintaining those strategies became exceedingly challenging. For instance, one participant drew parallels between the allure of drug use and the desire to gamble:

*“You really have to have extraordinary self-control. But already the fact that you get into this, you don’t really have self-control, you get along. You’re weak in some ways. The same as people who take alcohol, the same as people who smoke, the same as people who take drugs. So there is a weakness somewhere, there is a problem. If we were strong enough, we were, we say why do I take drugs to have I don’t even know what it gives, I’ve never taken, but I am, I never take it because I still have a responsibility towards it, but I have other weaknesses, you know, gambling, that’s a weakness.”*

*(Sabrina, age 47, PGSI 10)*

In situations where one was no longer able to self-regulate their behavior, participants reflected on the need for external intervention. Some participants noted the importance of onsite intervention facilitated by gambling service workers or other specialized staff trained to intervene in situations where a customer is demonstrating signs of gambling addiction. One participant described how an onsite intervention could take place:

*“That’s what I mean, there should be someone who would be responsible in the casino as I’m explaining to you, but again, is it possible to do this? A person who would say: “This person, I see you often, I see that you gamble too much, that it’s too much, you have problems, you’re there every night, you spend two thousand”, for example “You are too much, maybe you should stop””*

*(Gabriella, age 63, PGSI 7)*

Another participant explained that support for those with gambling problems should include guidance, especially guidance that would counter some of the promotional messaging disseminated by the gambling industry:

*“Because if, if you think all the time that you’re going to win, like the message, I’m telling you, on all the machines, you’re going to come more often because you’re going to say you’re going to win. But if there’s somebody that’s repeating to us, the same thing as the addicts, if there’s somebody that’s helping them all the time, all the time next door that’s explaining it to them, maybe they’re not going to be taken that badly. But there is no one to guide us, because we are sick somewhere, we have a problem. So we need to be brought back on a good path.”*

*(Sabrina, age 47, PGSI 10)*

## 4. Discussion

The aim of this paper was to explore self-control strategies used by individuals engaged in gambling activities, thus filling a gap in understanding the efficacy of and adherence to such strategies across different types of gamblers. The findings provided important insights into how individuals attempt to mitigate potential problems associated with gambling, revealing three main themes: limit-setting (encompassing setting time, frequency, or financial limits and limiting exposure to triggers), playing smart (involving mindful management of winnings and informed decision making), and the overall efficacy of these strategies. While these insights offered valuable contributions to gambling behavior, the study underscored their limitations, particularly in curbing compulsive gambling behaviors and especially among individuals with higher PGSI scores.

Limit-setting emerged as a prevalent approach among participants to regulate their gambling behavior, which involved restrictions on frequency, time, and spending. Participants demonstrated a proactive approach to self-regulation by imposing constraints on both the frequency and duration of their gambling sessions, reflecting a conscious effort to mitigate potential risks associated with gambling. Similar to previous findings, strategies included playing at certain intervals without explicit reasoning behind the chosen frequency, with the most common intervals being weekly or monthly sessions [9]. Adding to previously described limit-setting approaches, some participants also mentioned short-term abstinence as a strategy. In contrast to the prevalence of frequency limits and echoing previous research where few participants spontaneously discussed time strategies, time limits emerged as a less frequently mentioned strategy in this study, with only one participant bringing up this approach. These findings aligned with prior research emphasizing the significance of limit-setting in mitigating negative gambling outcomes [10] while offering insights into less-explored aspects, such as short-term abstinence.

Participants employed several strategies for setting spending limits, including budgeting, which has been reported in several previous studies [9,10,12]. Participants’ approaches to setting spending limits demonstrated individual differences based on personal circumstances and preferences. Participants tailored their budgets based on personal considerations, such as what they were willing to lose or deemed appropriate to spend, a facet less explored in the existing literature. Additionally, limiting access to money, another well-documented strategy [9,12], was discussed, with participants employing various tactics, like leaving money at home or refraining from bringing debit cards so as to avoid withdrawing more money. Notably, a newer aspect of this strategy emerged in our findings: reorienting money to limit access, such as depositing it into a separate bank account or investing it. The interaction between different limits revealed the connection between strategies. Breaching one limit often necessitated adjustments in others, highlighting the intricate dynamics at play in maintaining self-control when gambling. For instance, exceeding a predetermined spending limit prompted individuals to reconsider their frequency of gambling activities, illustrating how the non-observance of one limit can impact another [14]. However, the practical implementation of these limits posed challenges for individuals, underscoring the difficulties they encountered in adhering to their self-imposed boundaries in gambling activities [12,14].

Participants also recognized the influence of certain triggers on gambling behavior, employing strategies to limit access to specific game types, individuals, or alcohol. Our study uncovered participants’ proactive efforts to control these triggers, such as physically distancing themselves from locations with high-risk gambling forms or abstaining from alcohol during gambling sessions, which were identified as protective measures against impulsive behaviors and excessive losses. Flores-Pajot et al. elucidated the diverse array of triggers that can precipitate gambling behavior and the strategies individuals employ to mitigate their impact, echoing our study’s emphasis on participants’ proactive measures [9]. Additionally, Yi and colleagues’ study highlighted the significance of understanding and addressing triggers in gambling contexts, revealing how temptations or triggers can significantly affect individuals’ self-control, potentially leading to higher rates of limit violations [14]. Our findings emphasized the complex interplay between individual, environmental, and social factors, which is consistent with previous research [9,14].

Playing smart emerged as another main strategy among participants. This concept encompassed mindful management of winnings, use of knowledge and skill to minimize risks, and acknowledging the chance-based nature of gambling outcomes. Findings aligned with previous research, emphasizing strategic decision making [8,10] and self-regulation in gambling behavior [20], which included being mindful of when to stop in order to manage risk [9,10]. While previous research has explored informed decision making in responsible gambling [8,10], participants also emphasized understanding the rules of the game and employing logic-based strategies. In contrast to previous studies, our findings discussed the cognitive processes underlying gambling behaviors, such as educating oneself on game rules and strategically selecting pragmatic approaches that balance optimism and realism, all while acknowledging the unpredictability of gambling. This underscored the proactive role of skill and strategy in shaping gambling experiences, suggesting that individuals perceive their gambling experiences as being enriched through an informed approach. Participants stressed the need for self-awareness and realistic expectations, where they remained mindful of the nature of chance-based games while employing strategic approaches to maximize favorable outcomes and minimize risks.

While the above-noted self-control strategies were employed to regulate gambling behavior, this study revealed the challenges individuals face in effectively implementing these strategies. This aligns with previous research emphasizing the limitations of self-control strategies in mitigating the risks associated with gambling [12,14]. While Yi and colleagues discussed specific negative emotions and feelings experienced when self-imposed limits were violated, such as increased anxiety, participants in our study did not explicitly mention these emotions [14]. However, our findings revealed that participants did express feelings of disappointment and regret over their inability to maintain self-control. Despite a variety of self-control strategies reported—such as restricting access to funds or imposing time limits—most participants found themselves unable to maintain self-control. In particular, participants with higher PGSI scores articulated the challenges they faced in implementing these strategies, shedding light on the limitations of self-control strategies in curbing compulsive gambling behaviors and expressing feelings of disappointment and regret over their inability to do so.

These findings underline the need for public health policies that go beyond promoting individual responsibility and incorporate structural interventions within gambling environments. For example, policies could introduce proactive harm-reduction strategies in venues like casinos, such as mandating regular self-assessment tools to help individuals monitor their gambling behavior. Further research should assess the effectiveness of these interventions, particularly for individuals with higher PGSI scores, and their impact on reducing gambling-related harms. Additionally, longitudinal studies are needed to examine how these policy changes contribute to long-term behavioral change and sustained harm reduction.

Findings from this study also highlighted parallels between problem gambling and substance addiction, suggesting that addiction to gambling can be as severe as dependency on substances, such as cocaine. This finding is particularly significant, as it suggests that individuals struggling with gambling addiction may face similar difficulties in adhering to self-imposed limits as those with substance dependencies. Previous literature has acknowledged the limitations of self-imposed strategies in mitigating the risks associated with gambling [12,14]. By recognizing the parallels between gambling addiction and substance use addiction, our findings underscored the potential to draw upon models and approaches used in substance addiction interventions to guide gambling interventions. Such an approach may better address the multifaceted nature of gambling addiction and improve outcomes for individuals struggling with this disorder.

While participants used various self-control tactics, such as limit-setting and playing smart, their struggles in maintaining self-control, particularly among those with higher PGSI scores, underscored strategy limitations. These findings highlighted the need for future research to better understand the barriers to adherence and the effectiveness of self-control strategies for those participating in gambling activities. Moving forward, research could focus on innovative intervention approaches tailored to address these challenges and provide more effective external controls, thereby reducing the heavy reliance on self-control strategies that have significant limitations. Furthermore, the parallels drawn between problem gambling and substance addiction emphasized the possibility of drawing upon models and approaches used in substance addiction when developing gambling interventions. By integrating insights from research on self-control strategies into the design of comprehensive interventions, researchers can develop more effective harm minimization measures and support systems within both offline and online gambling environments. Ultimately, this study lays the groundwork for future research to advance our understanding of self-control in gambling and to inform the development of interventions that foster healthier gambling practices.

## 5. Limitations

Qualitative inquiry provides a deep understanding of complex human behavior, but the findings from this study are not generalizable. Although recruitment efforts resulted in a diverse sample in terms of age, gender, and PGSI scores, the primary aim of this qualitative research was not to generalize to the broader gambling population but to offer rich insights into participants’ lived experiences. The non-random sampling approach may have limited the diversity of perspectives, but it aligns with the exploratory nature of the study. Potential biases, such as self-selection and social desirability, could have influenced participants’ responses, although these are inherent to qualitative research and provide valuable reflections on the subjective experiences of individuals. Future research could expand the sample to include a wider range of participants, thus helping to capture additional nuances and variations in gambling experiences. Despite this, the recruitment efforts produced a diverse sample in terms of age, gender, and PGSI scores, which likely reflects some diversity in participants’ lived experiences. While recall bias and social desirability bias are potential limitations, probing techniques were used to clarify responses, and a trained interviewer created a safe environment for open dialogue. Because the data were collected for a related study with a different objective, this may limit the depth of the findings, although probing was employed to explore emerging themes around strategies. For instance, future research may benefit from a focused inquiry into the origin of these strategies, including whether they were self-taught or learned through peers or treatment professionals.

## 6. Conclusions

In conclusion, this study sheds light on the limitations of self-control as a strategy for preventing gambling problems, thus highlighting the challenges individuals face in effectively implementing them, which is in line with previous research findings [21]. Despite the multitude of strategies employed, adherence remains a significant obstacle, suggesting that promoting self-control alone may not suffice in curbing compulsive gambling behaviors. These findings hold broader implications for individuals, policymakers, and researchers in addressing problem gambling, emphasizing the need for comprehensive support systems and interventions within both offline and online gambling environments to mitigate gambling harm.

## Figures and Tables

**Table 1:** Demographics and gambling profile of participants (N = 30).

Age at Time of Interview	
Mean	50.5 years
Gender	
Male	20
Female	10
Marital Status	
Single	21
Divorced/separated	3
Living with a partner	4
Married	1
Widower	1
Employment Status	
Employed—full-time	11
Employed—part-time	4
Not employed	15
Education Level	
Secondary-level education or less	11
College-level education (partial or complete) or equivalent (vocational or technical school)	10
At least a Bachelor’s degree	8
Preferred not to answer	1
Annual Income Before Taxes	
Under CAD 20,000	10
CAD 20,000–49,999	13
Above CAD 50,000	3
Preferred not to answer	4
PGSI Score	
Non-problem gambling (0)	5
Low risk (1–2)	2
Moderate risk (3–7)	10
Problem gambling (8 or more)	13

## Data Availability

The data presented in this study are available upon request from the corresponding author. The data are not publicly available due to ethical restrictions.
